# The roles of different microRNAs in the regulation of cholesterol in viral hepatitis

**DOI:** 10.1186/s12964-023-01250-w

**Published:** 2023-09-14

**Authors:** Xuan Meng, Yeganeh Eslami, Ehsan Derafsh, Anwar Saihood, Nikoo Emtiazi, Saman Yasamineh, Omid Gholizadeh, Renzon Daniel Cosme Pecho

**Affiliations:** 1 Hepatobiliary Surgery Department, National Cancer Center/National Clinical Research Center for Cancer/Cancer Hospital, Chinese Academy of Medical Sciences and Peking Union Medical College, Beijing, 100021 China; 2 Jiangsu Center for the Collaboration and Innovation of Cancer Biotherapy, Cancer Institute, Xuzhou Medical College, Xuzhou, Jiangsu 221002 China; 3 Faculty of Medicine, Mazandaran University of Medical Sciences, Sari, Iran; 4 Windsor University, School of Medicine, St. Kitts, Canada; 5 Department of Microbiology, college of medicine, University of Al-Qadisiyah, Baqubah, Iraq; 6 Department of Pathology, Firoozgar Hospital, Iran University of Medical Sciences, Tehran, Iran; 7 Young Researchers and Elite Club, Tabriz Branch, Islamic Azad University, Tabriz, Iran; 8 Department of Bacteriology and Virology, Faculty of Medicine, Tabriz University of Medical Sciences, Tabriz, Iran; 9 Department of Biochemistry, UNIVERSIDAD SAN IGNACIO DE LOYOLA (USIL), Lima, Peru

**Keywords:** microRNAs, Cholesterol, Viral infections, Viral hepatitis

## Abstract

Cholesterol plays a significant role in stabilizing lipid or membrane rafts, which are specific cellular membrane structures. Cholesterol is involved in numerous cellular processes, including regulating virus entry into the host cell. Multiple viruses have been shown to rely on cholesterol for virus entry and/or morphogenesis. Research indicates that reprogramming of the host’s lipid metabolism is associated with hepatitis B virus (HBV) and hepatitis C virus (HCV) infections in the progression to severe liver disease for viruses that cause chronic hepatitis. Moreover, knowing the precise mode of viral interaction with target cells sheds light on viral pathogenesis and aids in the development of vaccines and therapeutic targets. As a result, the area of cholesterol-lowering therapy is quickly evolving and has many novel antiviral targets and medications. It has been shown that microRNAs (miRNAs) either directly or indirectly target the viral genome, preventing viral replication. Moreover, miRNAs have recently been shown to be strong post-transcriptional regulators of the genes involved in lipid metabolism, particularly those involved in cholesterol homeostasis. As important regulators of lipid homeostasis in several viral infections, miRNAs have recently come to light. In addition, multiple studies demonstrated that during viral infection, miRNAs modulate several enzymes in the mevalonate/cholesterol pathway. As cholesterol metabolism is essential to the life cycle of viral hepatitis and other viruses, a sophisticated understanding of miRNA regulation may contribute to the development of a novel anti-HCV treatment. The mechanisms underlying the effectiveness of miRNAs as cholesterol regulators against viral hepatitis are explored in this review.

Video Abstract

**Graphical Abstract:**

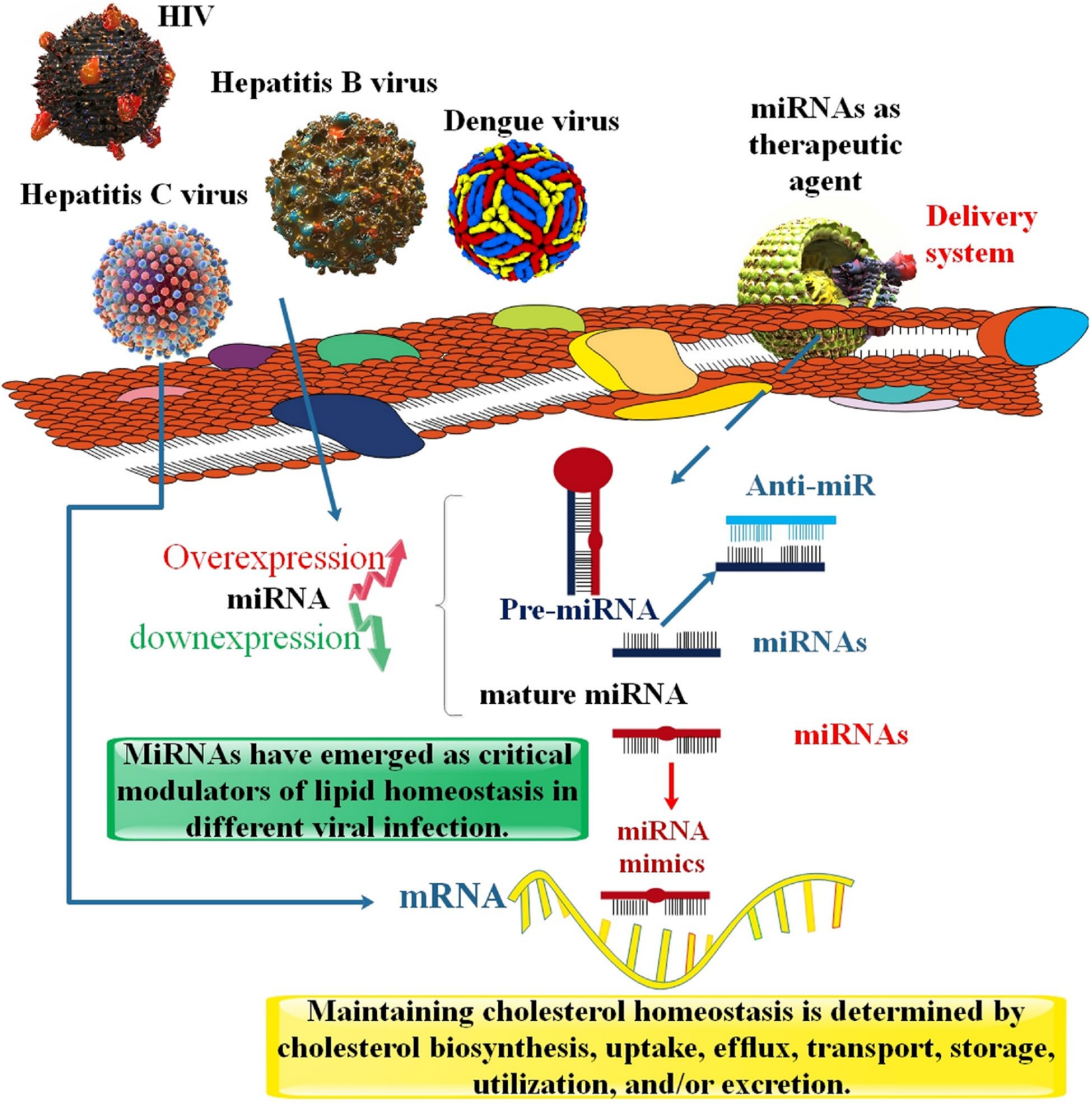

**Supplementary Information:**

The online version contains supplementary material available at 10.1186/s12964-023-01250-w.

## Introduction

One of the key elements of mammalian cellular membranes is cholesterol. It is necessary for the normal operation of membrane properties like permeability, fluidity, and organelle identity. It is an essential biomaterial for preserving membrane protein structures and regulating their functions. Moreover, practically all steroid hormones need cholesterol as a precursor [1, 2]. Elevated cholesterol is a hallmark of many metabolic and neurological diseases, including Alzheimer’s, cardiovascular disease, diabetes, viral infections, and chronic inflammation [3]. Cholesterol has been implicated in the replication of many different types of viruses, including filoviruses [4], hepatitis virus [5], coronavirus [6], pseudorabies virus [7], human immunodeficiency virus (HIV) [8], influenza virus [9], and chikungunya virus [10]. Both the virus and the infected host cell have a stake in cholesterol’s role in viral infection. As viruses lack internal membrane structures, the only thing that counts for a single virus particle is obtaining enough sterol from an infected cell to keep the bilayer of its envelope membrane intact. To leave the infected cell, the virus must first breach the plasma membrane, then express its genetic material, and finally construct new virions. Cholesterol may boost the infectivity of viruses by increasing and maintaining the local lipid bilayer bending that occurs during membrane fusion during the viral entrance of enveloped viruses [11]. From the perspective of the host cell, the pathogenic viral cycle causes dramatic alterations to the cell’s structure and metabolism. Pathological modifications are made to organelles, primarily the membrane-rich endoplasmic reticulum-Golgi complex (the so-called ERGIC), to form the replication-transcription organelle, the site of viral genome replication, and the sequestration of its biosynthetic machinery to serve the production of many viral particles under the control of the viral genome [12]. For two highly contagious enveloped viruses, influenza A virus (IAV) and SARS-CoV-2, endolysosomal cholesterol homeostasis may be an appropriate antiviral target [4, 13]. In most cases, they induce a mild form of hepatitis that resolves on its own. Rarely can these viruses cause persistent infections in healthy people. Nevertheless, acute and chronic hepatitis is caused by the hepatitis B virus (HBV), a double-stranded DNA virus from the family Hepadnaviridae; and the hepatitis C virus (HCV), a positive-sense single-stranded RNA virus from the family Flaviviridae. Liver fibrosis, cirrhosis, and, in many instances, hepatocellular cancer (HCC) are all long-term outcomes of chronic viral hepatitis [14]. Moreover, sections of structural proteins are localized at lipid-raft-like membrane structures inside cells, and cholesterol and sphingolipid on the HCV membrane play significant roles in viral internalization. Inhibitors of the sphingolipid biosynthesis pathway are the first and only drugs shown to effectively halt virus replication [15]. Inhibiting cholesterol production has been shown to diminish infections of many human viruses, including HBV and HCV, suggesting a central function for host cholesterol levels in viral infections. Hence, cholesterol-lowering medicines are offered as an approach to treat hepatitis and other viral illnesses by manipulating cholesterol production [16]. miRNAs have recently been shown to be powerful post-transcriptional regulators of lipid metabolism genes, including cholesterol homeostasis, in addition to the traditional transcriptional control of cholesterol metabolism (e.g., by SREBP and LXR) [17].

**Fig. 1:**
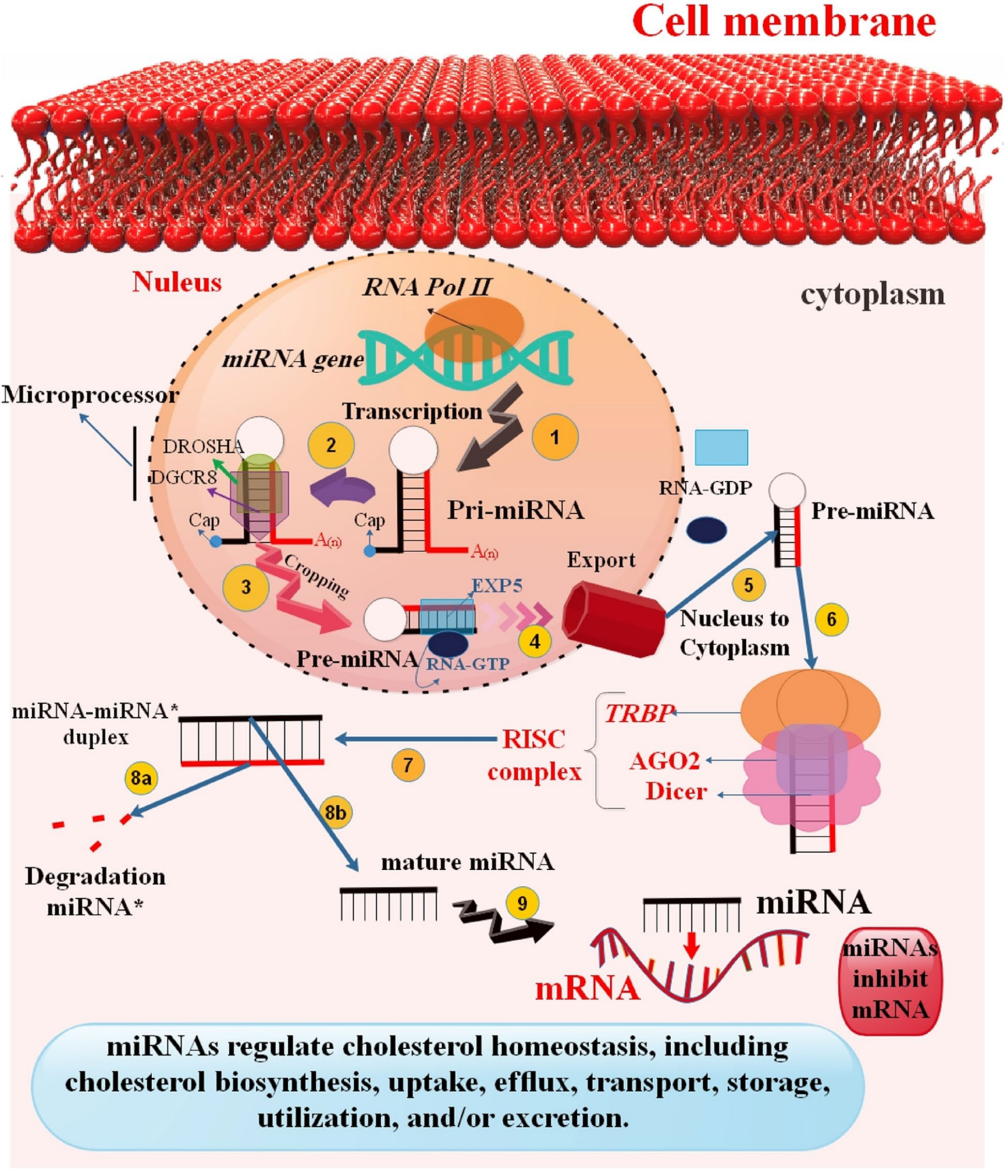
Biogenesis of miRNAs and Cholesterol Control. In a nutshell, miRNAs are first transcribed by RNA polymerase II (RNAP II), which then produces an initial transcript known as produced precursor miRNAs (pri-miRNA) [22, 23]. Following transcription, the common hairpin-loop secondary structure seen in pri-miRNAs is recognized and cleaved by the microprocessor complex (created by DGCR8 and Drosha). To create a double-stranded RNA, pre-miRNAs are transferred to the cytoplasm and then digested by the Dicer nuclease. The RNA-induced silencing complex (RISC), which utilizes the mature miRNA sequence to perform its regulatory role, is chosen through Ago2 [24–26]. Each miRNA may bind to a number of mRNAs involved in the transport, storage, utilization, and/or excretion of cholesterol as well as in its production, absorption, efflux, and other processes

miRNAs are a class of short, single-stranded, endogenous, highly conserved, non-coding RNAs with essential regulatory roles in a broad variety of cellular and physiological activities, including proliferation, differentiation, neoplastic transformation, and cell regeneration [18]. The expression of human protein-coding genes is fine-tuned by miRNAs, which recognize specific sequences in mRNA transcripts and direct the RNA interference silencing complex to sequester and destroy those transcripts. Because of the short length of base pairing and imprecise complementarity, mRNAs are often targeted by many miRNAs, and a single miRNA will target numerous mRNAs, generally clustered around a specific biological function. Since miRNAs may affect so many distinct cellular processes and pathways, their expression and control can promote or suppress a wide variety of pathogeneses [19]. As a result of viral infection, host miRNA expression levels shift. These miRNAs are up-or down-regulated in response to viral infection, and they govern viral replication, the innate immune response, and cell death by targeting the viral genome directly or indirectly [20]. In addition, alterations in the expression of the relevant miRNAs are strongly linked to metabolic diseases, and miRNAs have been demonstrated to influence critical proteins in cholesterol homeostasis. It has been shown via research that a single miRNA may operate as a target for numerous mRNAs in the 3′-untranslated region (UTR), and that a single mRNA molecule can be anticipated to be the target of many miRNAs. Thus, to properly maintain intracellular cholesterol homeostasis, miRNAs’ posttranscriptional regulation activity constitutes another crucial layer of complex regulatory networks, in addition to the regulatory layer of transcription factors and co-activators [21] **(**Fig. 1**)**. By investigating how viruses regulate host processes via miRNA, we may learn more about these relationships and identify possible novel treatment targets. Consequently, the purpose of this review is to highlight the role of miRNAs in regulating viral reproduction by influencing cholesterol levels.

### MiRNAs in viral infection

Each miRNA can bind to several mRNAs. MiRNAs function to decrease protein synthesis of protein-coding genes by inhibiting the translation of targeted mRNA or by mRNA degradation. Additionally, miRNAs can activate the translation of specific mRNAs, cycling between translation suppression and activation in tandem with the cell cycle. In addition, miRNAs have been demonstrated to play a critical purpose in various biological functions, such as metabolism, immunity, cell growth, apoptosis, differentiation, and signal transmission. The dysregulation of miRNAs in sickness settings might be employed as prospective curatives by either miRNA replacement therapy employing miRNA mimics or inhibition of miRNA function via antagomiRs [27]. Antisense oligonucleotides (ASO) against miRNA are a promising new area of medicinal nucleic acid research [28]. Compared to protein-based medications and even plasmid-DNA-based gene therapy, miRNAs have fewer adverse effects and are less immunogenic. There are currently two ways to employ miRNAs for therapeutic purposes: (1) to block oncogenic miRNAs using miRNA antagonists like antimiRs or antagomiRs, and (2) to introduce tumor suppressor miRNAs using either synthetic miRNA mimics or stable, vector-based transfection of miRNA-coding genes. MiRNA antagonists are single-stranded-RNA molecules of 21–23 nucleotides that work with miRNAs by complementary base pairing [20, 29].

It is currently thought that viruses, which make use of a variety of host gene expression mechanisms, can encode miRNAs in their genomes. Viral miRNAs (v-miRNAs) have been shown to play several significant functions in studies during the last ten years. In addition, several host-encoded miRNAs may be able to regulate viral replication by interacting with viral RNAs’ target regions [30]. EVs containing miRNAs in viral infections may be used as functional mediators of several biological processes involving cell-to-cell communication as well as helpful indicators of illness states. Moreover, they play a significant role in the control of immunological responses. Understanding the role of miRNAs in this setting might thus aid in the identification of relevant biomarkers and/or possible treatment targets [31]. In response to viral infection, host miRNA expression levels fluctuate. These altered miRNAs enhance or suppress the innate immune response and cell death during viral infection, and they directly or indirectly target the viral genome to govern viral replication [32]. For instance, in response to influenza virus stimulation, host miRNA expression is changed. During viral infection, these dysregulated miRNAs activate or repress innate immune responses, cell death, and viral multiplication by directly or indirectly targeting viral genes [32]. Changes in the expression profile of the miRNAs in epithelial cells may contribute to the pathogenesis of both acute and chronic respiratory diseases. The immune response against respiratory viruses, such as the human rhinovirus, influenza virus, human metapneumovirus, human coronavirus, and respiratory syncytial virus, is associated with the altered expression of several miRNAs [33]. Recent research has shown that miRNAs can regulate viral protein synthesis, replication, and pathogenicity by focusing on RNA virus genomes. In addition, the overexpression or downregulation of host cell miRNAs during RNA virus infection might result in subsequent alterations to the infected cell’s transcriptome that serve to further propagate the infection [34].

v-miRNAs are small interfering RNAs that are encoded by DNA and RNA viruses and play a role in immunological dysregulation, viral generation, and disease pathogenesis. miRNAs expressed by viruses may play a significant part in this process since they are perfect and non-immunogenic tools for viruses, allowing them to control the expression of their own and their host’s genes. As they implement a variety of immune evasion methods, V-miRNAs seem to play a pivotal role in viral persistence and spread. V-miRNAs and host miRNAs can both regulate the expression of multiple host- and virus-derived transcripts. Using v-miRNA orthologues of cellular miRNAs, which share a seed sequence and so control the same targets, is an attractive idea [35, 36] (Table 1).

**Table 1:** miRNAs function in viral infections

Viral infections	miRNAs	miRNA function in viral infection	Ref
HBV	miR-155	Zinc fingers and homeoboxes 2 (ZHX2) levels were lowered by miR-155 overexpression through miR-155 seed sites in the ZHX2 3′UTR, but ZHX2 levels were raised by miR-155 inhibition. Therefore, data provide a potential treatment for HBV-related HCC by suggesting that HBV’s HCC-promoting features may involve silencing of ZHX2 through a miR-155-dependent route.	[37]
HBV	miR-122	By downregulating the Gld2 gene, HBV decreased the levels of miR-122, which may indicate a novel method for HBV to control the production of miRNAs. As a consequence of the Gld2 protein being downregulated by the HBV X (HBx) protein, the amount of miR-122 is lowered.	[38]
HAV	hsa-miR-146a-5p	hepatitis A virus (HAV) partly interferes with RIG-I/MDA5-mediated IFN-I signaling by cleaving TNF receptor-associated factor 6 (TRAF6), an important adaptor protein, through hsa-miR-146a-5p.	[39]
HCV	miR-122	Two locations in the 5’ UTR of the HCV genome are targeted by miR-122, which thus encourages viral RNA accumulation.	[40]
HCV	miR-199a∗	In two cell lines harboring HCV replicons (replicon cells), overexpression of miR-199a_*_ suppressed HCV genome replication. In contrast, suppression of miRNA by a particular ASO sped up viral multiplication. When miR-199a was overexpressed in HCV replicon cells, replicon RNA was deposited in RISC.	[41]
HEV	miR-214	Direct interactions between miR-214 and HEV RNA promote HEV replication and genome translation. The expression of protein C, the thrombin-negative regulator, is suppressed by miR-214. HEV ORF3, another viral component, similarly improves intracellular active thrombin levels. Furthermore, miR-214 specifically targets 2′-5′-oligoadenylate synthetase, an antiviral host factor.	[42]
SARS-CoV-2	miR-2392	It is essential for miR-2392 to drive downstream inhibition of mitochondrial gene expression, increase inflammation, glycolysis, and hypoxia, as well as support several COVID-19 infections-related symptoms. COVID-19 symptoms in the host are accompanied by overexpression of miR-2392.	[43]
HIV-1	miR-132	miR-132 is shown to be substantially increased after CD4 + T cell activation, and studies in the Jurkat CD4 + T cell line demonstrate that miR-132 promotes viral propagation. MeCP2, a miR-132 target, is upregulated, which boosts HIV-1 replication.	[44]
HIV-1	miR-29a	miR-29a suppresses viral replication by binding to and silencing a region in the 3′UTR of viral mRNAs. A cytokine-microRNA (i.e., IL-21/miR-29a) route was discovered by studying its effects on HIV-1 replication in vivo. MiR-29a has been shown to have a significant role in HIV-1 proliferation and latency.	[45]
Dengue virus	miR-548 g-3p	It was revealed that miR-548 g-3p inhibited DENV 1, 2, 3, and 4 replication and also inhibited the production of viral proteins by interfering with DENV translation.	[46]
Dengue virus	miR-21	miR-21 increases the replication of DENV 2. To study the role of miR-21 in DENV infection, we co-administered AMO-21 and a peptide nucleic acid-21 (PNA-21) construct having a nucleotide sequence complementary to AMO-21, and then infected the animals with DENV 2.	[47]

### Cholesterol in viral infection

Cholesterol has essential roles in the plasma membrane, where it helps to organize the biophysical properties of lipids and proteins. It has been shown that cholesterol has a role in the entry and/or morphogenesis of several different viruses [48]. The presence of cholesterol is crucial to the entrance mechanism of many enveloped viruses. Researchers discovered that the infectivity of HBV relies only on virion-bound cholesterol, making it distinct from HIV and other retroviruses while being similar to the influenza virus [49]. It has also been hypothesized that HCV RNA replication takes place on a cholesterol-dependent membrane structure called a lipid raft. Cholesterol-rich membrane rafts have been demonstrated to facilitate DEN-mediated viral entry and to initiate flavivirus-induced Akt phosphorylation activation throughout the flavivirus life cycle. Since cholesterol dramatically inhibited JEV and DEN-2 infectivity during the viral adsorption phase, it had a major impact on the first stage of the flavivirus life cycle. Cholesterol acted as a barrier to the entrance of flaviviruses, most likely during the fusion and RNA uncoating stages [11, 50]. Niemann-Pick C2 (NPC2) and Niemann-Pick C1 (NPC1), two highly conserved gene products, mediate the subsequent stage in the export of cholesterol from the lysosome. NPC1 is essential for cholesterol homeostasis inside cells by facilitating its export from endolysosomes [51]. beforetoHIV-1 and HAV both rely on the NPC1-mediated intracellular cholesterol transport pathway for successful infection. The NPC1-mediated intracellular cholesterol transport pathway is essential for the successful infection of HIV-1 and HAV [52–54].

Previous research has shown that 25-hydroxycholesterol (25HC), the enzymatic byproduct of cholesterol 25-hydroxylase (CH25H), (Another name for it is cholesterol 25-monooxygenase), has potent broad-spectrum antiviral action. Cholesterol 25-hydroxylase (CH25H) is mostly found in the endoplasmic reticulum (ER) and Golgi apparatus, where it catalyzes the oxidation of cholesterol to 25HC. Lipid metabolism, antiviral mechanisms, inflammatory response, cell survival, and others are only some of the many biological processes that have been linked to CH25H [55]. In particular, prior research has proven that 25HC has antiviral action against several viruses, including vesicular stomatitis virus, herpes simplex virus, murine hepatitis virus 68, ebola virus, Rift Valley fever virus, tick-borne encephalitis virus strain RSSE, HCV, Nipah viruses. Zika, SARS-CoV2, and other highly infectious viruses [56–59]. Mevalonate diphosphate decarboxylase (MVD) and 3-hydroxy-3-methylglutaryl coenzyme A (HMG-CoA) reductase are two intermediate-stage biosynthetic enzymes that are essential for viral replication [60, 61]. The biosynthetic enzyme DHCR24 produces cholesterol, which is a significant part of lipid rafts and is thought to be crucial to HCV replication. HCV infection increased the expression of DHCR24, an important host component that is crucial for HCV replication. A brand-new target for anti-HCV medications may be DHCR24 [62].

### miRNA regulation of cholesterol metabolism and hemostasis

**Fig. 2:**
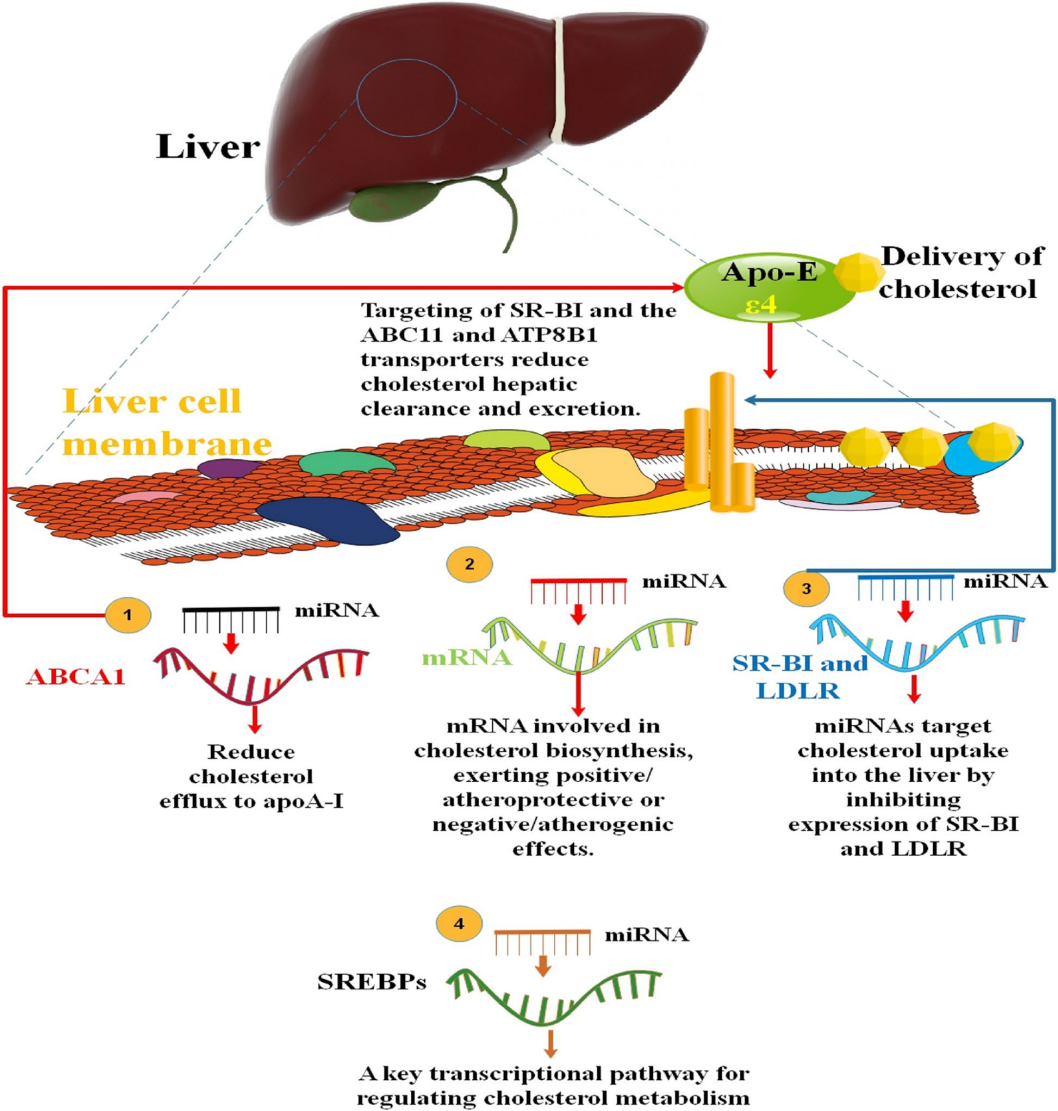
Reverse cholesterol transport and miRNAs in cholesterol homeostasis. MiRNAs exert either positive/atheroprotective or negative/atherogenic effects in the liver by directing their attention to key mediators involved in cholesterol production. These miRNAs suppress the target genes’ mRNA and protein expression, as stated. A variety of miRNAs in the liver target ATP-binding cassette transporter A1 (ABCA1) to decrease cholesterol efflux to lipid-poor apolipoprotein A-I (apoA-I), which produces nascent HDL. Scavenger receptor B-I (SR-BI) specifically binds to HDL’s miRNA and cholesterol cargo upon return to the liver, where excess cholesterol is discharged into the bile. By preventing the production of the cholesterol transporters scavenger receptor BI (SR-BI) and LDLR, miRNAs aim to reduce the absorption of cholesterol into the liver. SR-BI, ABC11, and ATP8B1 transporter inhibition decrease the hepatic clearance and excretion of cholesterol [72, 73]

Cholesterol production, absorption, efflux, transport, storage, utilization, and/or excretion have a role in maintaining cholesterol homeostasis. The many regulatory pathways should accurately govern every process. Numerous therapies have been created to decrease cholesterol by preventing cholesterol production and absorption or increasing cholesterol utilization and excretion. These strategies are based on the management of cholesterol homeostasis [63]. At the cellular level, cholesterol metabolism is strictly controlled. Recent research has shown that miRNAs are powerful post-transcriptional regulators of genes involved in lipid metabolism, including cholesterol homeostasis, in addition to the traditional transcriptional regulators of cholesterol metabolism (such as sterol regulatory element-binding protein and liver X receptor) [17, 64]. It has become clear that microRNAs play a crucial role in controlling circulating levels of lipoproteins. Recent studies have revealed that miRNAs play a key role in regulating the levels of atherogenic low-density lipoprotein (LDL)-cholesterol by acting on genes involved in very low-density lipoprotein (VLDL) secretion, cholesterol biosynthesis, and hepatic LDL receptor (LDLR) expression after transcription has already taken place. For example, the first miRNAs to be proven to affect plasma LDL-cholesterol (LDL-C) in vivo were miR-122 and miR-30c, both of which are found mostly in the liver and which function by regulating VLDL secretion and cholesterol production [65]. It has been established that miRNAs control critical proteins involved in cholesterol homeostasis and that abnormal expression of the relevant miRNAs is strongly linked to metabolic diseases. Sterol regulatory element-binding proteins (SREBPs) are involved in a crucial transcriptional pathway that controls cholesterol metabolism. Recent studies in mice and humans have shown that SREBP-2 directly stimulates the production of the miR-183/96/182 operon in the liver. SREBP-2 supports polycistronic transcription of the three miRs by interacting with a conserved binding site (E-box) at the TSS for the miR-183/96/182 transcription unit. MiR-182 regulates SREBP activity for cholesterol homeostasis by targeting FBXW7, while miR-96 regulates SREBP activity by targeting INSIG2 [21, 66, 67]. Some microRNAs, like miR-34a, regulate sirtuins and thus contribute to hepatic steatosis; others, like miR-33, regulate ATP-binding cassette transporters and thus regulate cholesterol flux; others, like miR-30, repress microsomal triglyceride (TG) transfer protein and thus regulate lipoprotein secretion; and still others, like miR-29, fine-tune the FOXA2-controlled gene network governing [68–71] (Fig. 2).

### miRNAs regulation of cholesterol in viral hepatitis

Hepatitis caused by viruses is an inflammatory disorder of the liver. Hepatitis viruses are the most prevalent cause of viral hepatitis, while infections with other viruses may also lead to liver inflammation. Five hepatitis viruses with unique genetic histories have been discovered. Hepatitis A virus (HAV), a member of the Picornaviridae; and hepatitis E virus (HEV), a member of the Hepeviridae, are two of the five viruses that may be spread by fecal–oral. Both are prevalent in low-income nations. In most cases, they induce a mild form of hepatitis that resolves independently. In immunocompromised individuals, chronic infections with these viruses are very unusual. HBV is a member of the family Hepadnaviridae, and HCV is a member of the family Flaviviridae. Both cause acute and chronic hepatitis. Liver fibrosis, cirrhosis, and, in many instances, hepatocellular carcinoma (HCC), are all long-term consequences of chronic viral hepatitis [16, 74, 75]. Both the hepatocellular (liver) phases of HCV entrance, replication, and assembly, as well as the circulating (blood) stages, when complex lipoviral particles (LVP) are formed, include lipids. Chronic HCV (CHC) infection results in abnormal lipid metabolism and is linked to reduced blood levels of LDL cholesterol and hepatic steatosis, both of which clear up after effective antiviral medication [76]. Increased cellular TG and cholesterol accumulation are two examples of how HCV-induced modifications of lipid metabolism aid viral proliferation. In addition, lipoprotein receptors and cholesterol have been linked to HCV entrance. Components of the VLDL pathway are also used in viral particle formation and secretion. Bezafibrate, a PPAR- agonist, may counteract the miR-27b-induced increase in lipid accumulation in Huh7 cells. One unexplored mechanism by which HCV causes hepatic steatosis is the downregulation of PPAR-signaling by miR-27b [77].

**Fig. 3:**
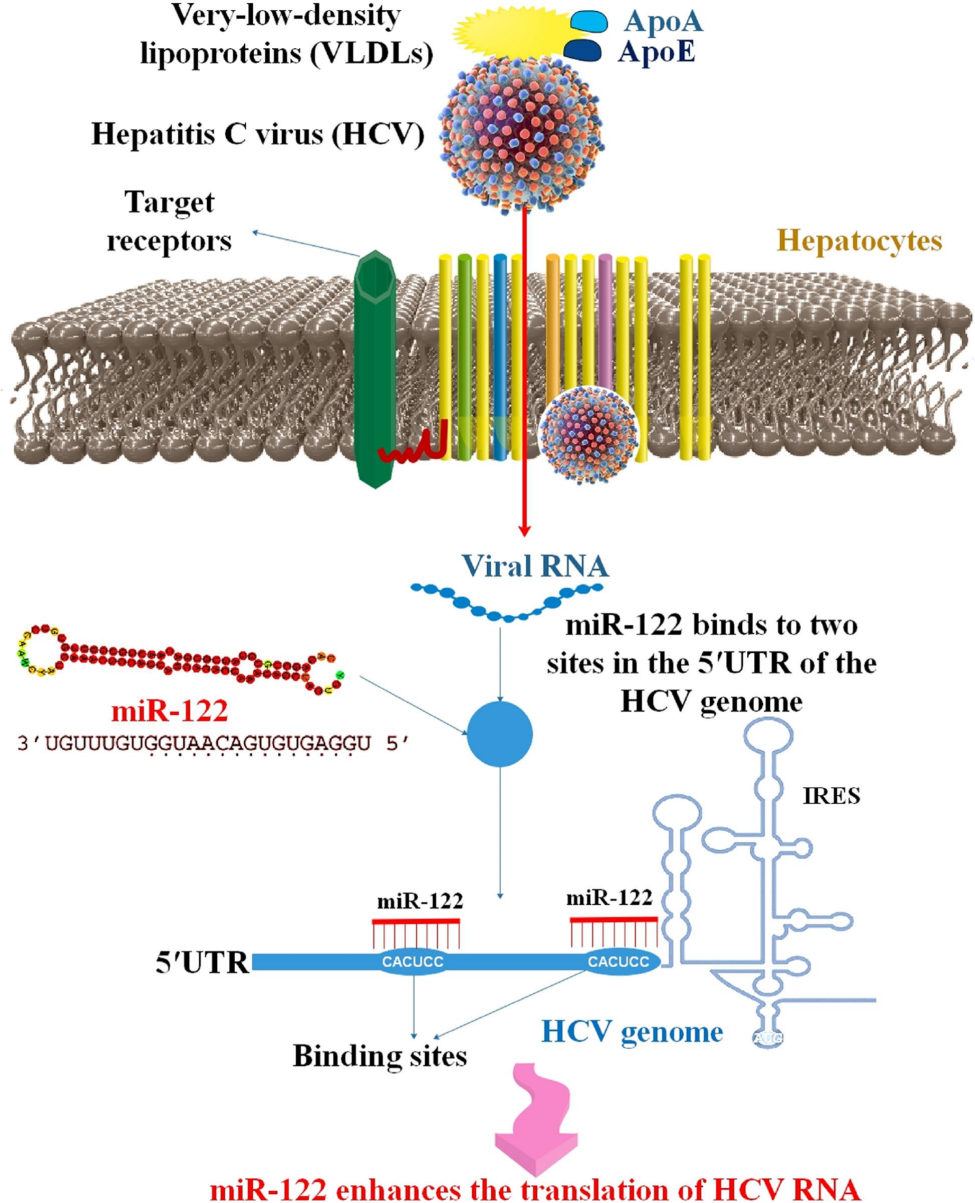
miR-122’s role in HCV. Liver-specific miR-122 binds to two sites in the 5′UTR of the HCV genome and increases its translation and replication. Direct effect, caused by miR-122 targeting of the HCV RNA; indirect impact, caused by downregulation of HMOX1, which has been shown to prevent HCV replication. HCV RNA abundance significantly decreased as a result of miR-122. When miR-122 binds to the HCV genome’s 5′ UTR, ribosome interaction with the viral RNA is improved. Putative effectors of miR-122-mediated metabolic regulation have been proposed to include AMP-activated protein kinase (APK) and circadian metabolic regulators of the PPAR family [86–89]

Highly dysregulated miR-27 significantly inhibits cholesterol production in CHC, chronic hepatitis B (CHB), and HCC. This is achieved in part through the regulation of the gene encoding the rate-limiting enzyme HMG-Co reductase (HMGCR). miR-27 and miR-21 are potential therapeutic targets for hypercholesterolemia [78]. The HCV core protein acted as a strong regulator of miR-185-5p expression. Furthermore, both the mRNA and protein levels of SREBP2 were suppressed when miR-185-5p was overexpressed. SREBP2 protein expression was increased by miR-185-5p suppression. The HCV core protein regulated the expression of SREBP2 through miR-185-5p [79]. Recently, it was shown that certain viral infections altered the immune-associated miR-146a, which is widely prevalent in peripheral blood mononuclear cells (PBMCs). The goal of the research was to examine how HCV infection affected miR-146a expression in PBMCs both in vivo and in vitro, as well as to determine if a change in miR-146a may affect the intracellular cholesterol level in PBMCs. Materials were drawn from blood samples taken from 72 CHC patients and 42 healthy individuals. In addition to determining the HCV genotype and interferon (IFN)-α concentration in sera, PBMCs were also tested for HCV RNA, intracellular cholesterol level, and miR-146a expression. Changes in the expression of miR-146a, intracellular cholesterol, and IFN-α in the sera of genotype 1 HCV-infected patients as compared to healthy donors. Additionally, miR-146a expression and intracellular cholesterol levels in cultured PBMCs were considerably lower in CHC patients than in healthy donors. Blocking miR-146a expression in CHC patients’ PBMCs in vitro significantly reduced intracellular cholesterol expression. Under these circumstances, there was a positive correlation between miR-146a expression and intracellular cholesterol level. These data point to a possible link between miR-146a expression changes in PBMCs and the deregulation of cholesterol production seen in genotype 1 HCV infection [80]. miR-122, a unique miRNA that directly regulates the HCV life cycle. miR-122 antagonists drastically lowered HCV titers in HCV-infected chimps and humans, making this miRNA a leading contender for the first miRNA-based treatment to get approval [81]. Although miR-122’s usual job is to control cholesterol and fatty acid production, it also promotes HCV RNA accumulation by interacting with two locations (site 1 and site 2) at the 5’ terminal of the HCV genome [82] (Fig. 3). One research was conducted to learn how miR-122 expression and cholesterol levels in PBMCs of CHC patients were affected by peginterferon alpha (pegIFN-α) and ribavirin therapies. After receiving antiviral therapy, PBMCs showed lower expression of miR-122 compared to their untreated counterparts. Concurrently, the amount of cholesterol in CHC patients’ PBMCs was greater six months after therapy compared to pretreatment levels. This suggests that one antiviral impact of pegIFN-α/ribavirin therapy for CHC patients is a reduction in miR-122 expression in PBMCs [83]. The expression of SREBP-1c and CAV1 is strongly upregulated in response to miR-29a induction, leading to an increase in lipid droplets (LDs) and TG. However, HCV RNA levels in Huh-7 cells were reduced when their production of miR-29a was induced [84]. The X protein of HBV (HBx) is an important contributor to the progression of HCC. Hepatocarcinogenesis has been linked to lipid metabolic abnormalities. In hepatoma cells, HBx inhibited miR-205. The potential aberrant lipid metabolism may be related to the HBx reduction in miR-205. In clinical HCC tissues, miR-205 expression levels were inversely correlated with those of acyl-CoA synthetase long-chain family member 4 (ACSL4). MiR-205 (or Triacsin C, an inhibitor of ACSL4) suppressed HBx’s ability to raise cellular cholesterol levels (a metabolite of ACSL4) in hepatoma cells. On the other hand, anti-miR-205 may elevate cellular cholesterol levels [85] (Table 2).

**Table 2:** miRNA as a cholesterol regulation method in viral hepatitis

Viral infections	miRNAs	miRNA regulate cholesterol in viral infection	Ref
HBV	miR-205	Clinical HCC tissues showed an inverse correlation between ACSL4 and miR-205 expression levels. MiR-205 inhibited HBx’s ability to raise cellular cholesterol, a byproduct of ACSL4, in hepatoma cells. On the other hand, anti-miR-205 may elevate cellular cholesterol levels.	[85]
HBV and HCV	miR-27	The gene encoding the rate-limiting enzyme in cholesterol production, HMGCR, is controlled by miR-27, which is considerably downregulated in CHC, CHB, and HCC.	[78]
HCV	miR-185-5p	SREBP2 mRNA and protein expression were suppressed when miR-185-5p was overexpressed. SREBP2 protein expression was increased by miR-185-5p suppression. HCV core protein mediated the effects of miR-185-5p on SREBP2 expression.	[79]
HCV	miR-146a	Expression of miR-146a and intracellular cholesterol in PBMCs, as well as IFN concentration in sera, differ between genotype 1, HCV-infected patients, and healthy donors.	[80]
HCV	miR-122	In comparison to the pretreatment condition, the level of miR-122 expression in the PBMCs dropped following the antiviral therapy. In addition, six months after therapy, the amount of cholesterol in the PBMCs of CHC patients was greater than it was prior to treatment. Therefore, it would appear that one of the antiviral effects of the pegIFN-alpha/ribavirin therapies is the reduction of miR-122 expression in the PBMCs of CHC patients.	[83]

### Limitation and advantages of use of miRNAs as therapeutic agents

Since viral infections are constantly evolving and developing new phenotypic features to capitalize on shifting host and environmental conditions, they represent serious difficulties for public health and are unlikely to be eradicated in the near future. There are currently no particular medications available to treat people who have contracted the majority of these numerous emerging and re-emerging viruses. Therefore, the creation and confirmation of efficient antivirals are urgently required. In light of these considerations, as well as the cost-benefit analysis for developing unique medications for each virus and the issue of the selection of drug-resistant mutants, new strategies are concentrated on the identification of broad-spectrum drugs targeting virally shared infection-initiating mechanisms [48]. The unique layer of the network that miRNAs provide for the control of genes gives them considerable potential as both a brand-new class of therapeutic targets and a formidable instrument for intervention. In this context, it has been demonstrated that synthesized RNAs that include the miRNA binding sites operate as a “decoy” or “miRNA sponge” to block the function of particular miRNAs. On the other hand, particular miRNAs have been restored or overexpressed using miRNA expression vectors to have a long-lasting impact. Additionally, experimental verification of double-stranded miRNA mimics for temporary replacement has been made [90]. Throughout the history of evolution, viruses have developed very complex strategies to take advantage of the biosynthetic machinery of host cells and avoid cellular defenses. Recent developments in research have shown that RNA-silencing pathways controlled by miRNA are also involved in the complicated interaction between viruses and host cells [91]. The nuanced roles that v-miRNAs play in extending the life of infected cells, thwarting the immune system, and controlling the transition to lytic infection are some of the activities that are well understood. Notably, all of these processes play a crucial role in chronic infections [92]. The use of miR-122 inhibitors in the treatment of HCV infection is one fascinating example of a miRNA-based antiviral medicine. A phase 2a trial was carried out by Janssen et al. (2013) in chronic HCV-infected individuals who got injections of Miravirsen every five weeks. The amount of HCV RNA decreased over time and in a dose-dependent manner as a consequence of the therapy. ASO for miR-122 which is N-acetylgalactosamine conjugated, known as RG-101, was also created to boost miR-122 sequestration by enhancing delivery in hepatocytes. In a phase 1B experiment, RG-101 therapy significantly reduced viral loads in all patients within 4 weeks of treatment [93–95]. Additionally, compared to protein-based medications and even plasmid-DNA-based gene therapy, miRNAs have fewer harmful side effects and are less immunogenic [86].

The use of miRNA as an anti-infective in clinical settings faces several significant obstacles. Their off-target consequences are miRNA treatments’ biggest drawback. This could be the result of non-specific interactions between miRNA and partly complementary mRNAs, which have significant adverse consequences on the host [96]. In addition, small molecule inhibitors have also had a poor track record in clinical trials due to issues such as tissue distribution, dosage optimization, and toxicity. However, the pleiotropic nature of miRNAs poses a significant obstacle to its treatment strategy. However, miRNAs may be chemically altered throughout the engineering process to increase stability and target specificity. The therapeutic method based on short RNAs has important advantages such as high specificity, controllable off-target effects, and the ability to avoid the side effects of targeting other mRNAs or protein functions. Although antisense oligos or siRNA-based pharmaceuticals make up the majority of the oligonucleotide/nucleic acid-based medicines, the effectiveness of the miRNA-based strategy has not yet been shown [97]. Furthermore, the presence of RNases, which can swiftly destroy miRNAs, may make it difficult for them to reach infected cells. Enhancing the delivery strategy of miRNAs to specifically target targets at the cellular or even subcellular levels, might be partly resolved. The use of nanoparticles, viral delivery methods, high-density lipoproteins, niosomes, liposomes, or exosomes, which may ease their distribution to host cells, have all been used to address this issue from various angles [27, 98, 99].

## Conclusion

Both the virus’s and the host cell’s perspectives on cholesterol in viral infection are possible. A virus particle’s capacity to receive the necessary quantity of sterol from the infected cell to maintain the structure of a single macromolecular assembly—its envelope membrane bilayer—is all that counts since it lacks interior membrane structures. Thus, broad-spectrum antivirals may facilitate better health outcomes for patients and quicker viral infection management. miRNAs, short RNAs that affect gene expression posttranscriptionally, have emerged as important modulators of lipid homeostasis. These findings bring to light the nuanced nature of lipid homeostasis and the crucial part played by miRNAs in the regulation of this process, suggesting novel approaches to the treatment of viral infections. The significance of miRNAs in controlling cholesterol homeostasis, especially against the backdrop of cirrhosis and the chronic inflammation prevalent in viral hepatitis, should be further explored in future studies. In the future, it may be able to cure HCV infection or at least significantly reduce liver pathogenesis by designing and delivering particular combinations of miRNA antagonists and mimics. The future of such treatment approaches and our knowledge of viral infection processes rely on a precise mapping and study of these signaling functions and bioactivities, many of which are harbored by cholesterol in particular. More research into the role of miRNAs in cholesterol metabolism and homeostasis across a variety of viruses is needed to address the method’s current shortcomings and advance its potential.

## Data Availability

Not applicable.
