# Correction: The roles of different microRNAs in the regulation of cholesterol in viral hepatitis

**DOI:** 10.1186/s12964-023-01325-8

**Published:** 2023-09-21

**Authors:** Xuan Meng, Yeganeh Eslami, Ehsan Derafsh, Anwar Saihood, Nikoo Emtiazi, Saman Yasamineh, Omid Gholizadeh, Renzon Daniel Cosme Pecho

**Affiliations:** 1 Hepatobiliary Surgery Department, National Cancer Center/National Clinical Research Center for Cancer/Cancer Hospital, Chinese Academy of Medical Sciences and Peking Union Medical College, Beijing, 100021 China; 2 Jiangsu Center for the Collaboration and Innovation of Cancer Biotherapy, Cancer Institute, Xuzhou Medical College, Xuzhou, 221002 Jiangsu China; 3 Faculty of Medicine, Mazandaran University of Medical Sciences, Sari, Iran; 4 Windsor University, School of Medicine, St. Kitts, Canada; 5 Department of Microbiology, College of Medicine, University of Al-Qadisiyah, Baqubah, Iraq; 6 Department of Pathology, Firoozgar Hospital, Iran University of Medical Sciences, Tehran, Iran; 7 Young Researchers and Elite Club, Tabriz Branch, Islamic Azad University, Tabriz, Iran; 8 Department of Bacteriology and Virology, Faculty of Medicine, Tabriz University of Medical Sciences, Tabriz, Iran; 9 Department of Biochemistry, UNIVERSIDAD SAN IGNACIO DE LOYOLA (USIL), Lima, Peru

**Correction: Cell Commun Signal 21, 231 (2023)**

**https://doi.org/10.1186/s12964-023-01250-w**

Following publication of the original article [1], the authors identified a typesetting error pertaining to the name of the author Renzon Daniel Cosme Pecho. The author’s name was mistakenly tagged as two names. This has been amended in this correction article and the original article [1] has been corrected.
